# A Successful Case of Cæsarian Section

**Published:** 1877-04

**Authors:** 


A Successful Case of Cæsarian Section, in which Silver Sutures were Used for the Uterine Wound.—Dr. Lungren, of Toledo, U. S., relates the case of a patient, aged twenty-nine, the subject of severe rachitic deformity of the pelvis. She married at the age of twenty-one, and became pregnant four times. The first time the fœtus perished about the sixth month and was spontaneously expelled about a week after. The second time abortion was induced at the third month. The third time abortion occured from unknown causes at the fourth month. In her fourth pregnancy she came under observation at the seventh month. Dr. Lungren decided to induce premature labour, and commenced to do so by dilating the os with Molesworth's elastic dilators. This was done until the largest dilator was reached, and it was then found that there was an unexpected degree of contraction at the brim. The promontory of the sacrum reached into the centre of the cavity, resembling a fœtal head, and the maximum antero-posterior diameter did not exceed from two and a quarter to two and a half inches. The membranes not having been ruptured, it was resolved to abandon the induction of labour, and let matters go on to full term.

Labour commenced on May 8th, 1875. The operation of Cæsarian section was undertaken when the os uteri was dilated to about an inch, filled with the pouch of liquor amnii. A final examination having been made under chloroform, it was thought that embryotomy would be so dificult as to be very dangerous to the mother, while it would certainly sacrifice the child. The placenta was found to be attached at the anterior surface of the uterus, and was wounded before the incision in its walls was extended to a length of two inches. Upon this the membranes were ruptured by the vaginia, to prevent the liquor amnii from escaping into the peritoneal cavity. The uterine wound was then enlarged to a length of six inches, and the fœtus was extracted by the head. It was alive, and immediately began to cry. The placenta was removed directly after. At this moment profuse hæmorrhage occurred, but by grasping the uterus with both hands, some contraction was induced, and the bleeding partially checked. The edges of the uterine wound, however, gaped widely, and blood continued to escape through the opening. Four sutures of silver wire were then inserted through the uterine wall, passing just short of its mucous membrane. Each wire was twisted twice only, care being taken to bring the edges of the peritoneum in apposition, then cut short and the ends bent down in the wound. The hæmorrhage was then arrested. After sponging out the peritoneum, the abdominal wound was at once closed.

For the first twenty-four hours there was rather acute pain, but after the first day the pulse never rose above 80, nor temperature above 100·5°. Five weeks after the operation she was attending to her household duties and washing the household linen.—*Reported in Archives de Tocologies, January,* 1877.

